# Effect of nirmatrelvir/ritonavir (Paxlovid) on hospitalization among adults with COVID-19: An electronic health record-based target trial emulation from N3C

**DOI:** 10.1371/journal.pmed.1004493

**Published:** 2025-01-17

**Authors:** Abhishek Bhatia, Alexander J. Preiss, Xuya Xiao, M. Daniel Brannock, G. Caleb Alexander, Robert F. Chew, Hannah Davis, Megan Fitzgerald, Elaine Hill, Elizabeth P. Kelly, Hemalkumar B. Mehta, Charisse Madlock-Brown, Kenneth J. Wilkins, Christopher G. Chute, Melissa Haendel, Richard Moffitt, Emily R. Pfaff, the N3C Consortium

**Affiliations:** 1 University of North Carolina at Chapel Hill, Chapel Hill, North Carolina, United States of America; 2 RTI International, Durham, North Carolina, United States of America; 3 School of Medicine, Public Health, and Nursing, Johns Hopkins University, Baltimore, Maryland, United States of America; 4 Patient-Led Research Collaborative, New York, New York State, United States of America; 5 University of Rochester, Department of Public Health Sciences and Department of Economics, Rochester, New York State, United States of America; 6 University of Tennessee Health Science Center, Memphis, Tennessee, United States of America; 7 National Institute of Diabetes & Digestive & Kidney Diseases, Office of the Director, National Institutes of Health, Bethesda, Maryland, United States of America; 8 F. Edward Hébert School of Medicine, Department of Preventive Medicine & Biostatistics, Uniformed Services University of the Health Sciences, Bethesda, Maryland, United States of America; 9 Emory University, Atlanta, Georgia, United States of America; Washington University School of Medicine, UNITED STATES OF AMERICA

## Abstract

**Background:**

Nirmatrelvir with ritonavir (Paxlovid) is indicated for patients with Coronavirus Disease 2019 (COVID-19) who are at risk for progression to severe disease due to the presence of one or more risk factors. Millions of treatment courses have been prescribed in the United States alone. Paxlovid was highly effective at preventing hospitalization and death in clinical trials. Several studies have found a protective association in real-world data, but they variously used less recent study periods, correlational methods, and small, local cohorts. Their estimates also varied widely. The real-world effectiveness of Paxlovid remains uncertain, and it is unknown whether its effect is homogeneous across demographic strata. This study leverages electronic health record data in the National COVID Cohort Collaborative’s (N3C) repository to investigate disparities in Paxlovid treatment and to emulate a target trial assessing its effectiveness in reducing severe COVID-19 outcomes.

**Methods and findings:**

This target trial emulation used a cohort of 703,647 patients with COVID-19 seen at 34 clinical sites across the United States between April 1, 2022 and August 28, 2023. Treatment was defined as receipt of a Paxlovid prescription within 5 days of the patient’s COVID-19 index date (positive test or diagnosis). To emulate randomization, we used the clone-censor-weight technique with inverse probability of censoring weights to balance a set of covariates including sex, age, race and ethnicity, comorbidities, community well-being index (CWBI), prior healthcare utilization, month of COVID-19 index, and site of care provision. The primary outcome was hospitalization; death was a secondary outcome. We estimated that Paxlovid reduced the risk of hospitalization by 39% (95% confidence interval (CI) [36%, 41%]; *p* < 0.001), with an absolute risk reduction of 0.9 percentage points (95% CI [0.9, 1.0]; *p* < 0.001), and reduced the risk of death by 61% (95% CI [55%, 67%]; *p* < 0.001), with an absolute risk reduction of 0.2 percentage points (95% CI [0.1, 0.2]; *p* < 0.001). We also conducted stratified analyses by vaccination status and age group. Absolute risk reduction for hospitalization was similar among patients that were vaccinated and unvaccinate, but was much greater among patients aged 65+ years than among younger patients. We observed disparities in Paxlovid treatment, with lower rates among black and Hispanic or Latino patients, and within socially vulnerable communities. This study’s main limitation is that it estimates causal effects using observational data and could be biased by unmeasured confounding.

**Conclusions:**

In this study of Paxlovid’s real-world effectiveness, we observed that Paxlovid is effective at preventing hospitalization and death, including among vaccinated patients, and particularly among older patients. This remains true in the era of Severe Acute Respiratory Syndrome Coronavirus 2 (SARS-CoV-2) Omicron subvariants. However, disparities in Paxlovid treatment rates imply that the benefit of Paxlovid’s effectiveness is not equitably distributed.

## Abstract 2

Abhishek Bhatia and team estimate the real-world effectiveness of Paxlovid and examine treatment disparities using target trial emulation on a cohort of 703,647 patients from N3C’s electronic health record database.

## Author summary

**Why was this study done?:**

- Paxlovid is widely used to prevent severe Coronavirus Disease 2019 (COVID-19), but its real-world effectiveness is uncertain.
- Prior studies differed in their findings, and none of them used a large, nationally sampled, and recent cohort.
- It is unknown whether Paxlovid may be more or less effective in certain demographic groups.

**What did the researchers do and find?:**

- We used a cohort of 703,647 patients from the National Covid Cohort Collaborative’s (N3C’s) electronic health record database to estimate Paxlovid’s real-world effectiveness and investigate disparities in treatment.
- We used the target trial emulation technique to estimate the causal effect of Paxlovid treatment using observational data.
- We found that Paxlovid treatment reduced the risk of hospitalization by 39% **and the risk of death by 61%**.
- We found that Paxlovid reduced the risk **of hospitalization** by a similar **absolute** amount regardless of whether the patient was vaccinated prior to getting COVID-19.
- We found that Paxlovid reduced risk more among patients aged 65+ years than among younger patients.
- We found disparities in Paxlovid treatment, with lower rates among black and Hispanic or Latino patients, and within socially vulnerable communities.

**What do these findings mean?:**

- Paxlovid is effective at preventing severe COVID-19 and is effective against current variants.
- Fully vaccinated people may have a further reduced risk of severe outcomes by receiving Paxlovid.
- People aged 65 years or older may benefit the most from Paxlovid treatment.
- However, disparities in Paxlovid treatment rates imply that the benefit of Paxlovid’s effectiveness is not equitably distributed.
- This study’s main limitation is that it estimates causal effects, but it is not randomized like a clinical trial. Instead, we control for other variables which could bias the estimate, but if we missed some important variables, the estimates could be incorrect.

## Introduction

The Coronavirus Disease 2019 (COVID-19) pandemic has had a profound global impact, with over 775 million cases and 7 million deaths as of 24 April 2024 [1]. This crisis has been met with research and drug development efforts at unprecedented speed, resulting in a number of new treatments aimed at lessening the risk of progression to severe disease. One such treatment, Paxlovid, is a combination of nirmatrelvir, an inhibitor of the main Severe Acute Respiratory Syndrome Coronavirus 2 (SARS-CoV-2) protease, and ritonavir, a pharmacological booster. In December 2021, the United States Food and Drug Administration (FDA) issued an Emergency Use Authorization (EUA) for Paxlovid, enabling its prescription to high-risk SARS-CoV-2–positive patients aged 12 years and older [2]. The EUA was based on the phase II-III EPIC-HR (Evaluation of Protease Inhibition for Covid-19 in High-Risk Patients) trial, which reported an 88.9% reduction in the risk of COVID-related hospitalization or death among those who received Paxlovid compared to those who received placebo [3].

Since the EUA issuance, several studies have assessed the effectiveness of Paxlovid using real-world data. An electronic health record (EHR)-based study in the Kaiser Permanente Southern California health system found that fewer than 1% of patients treated with Paxlovid (*n* = 5,287) were hospitalized or seen in the emergency department within 5 to 15 days of the drug being dispensed, though this was not compared with an untreated group [4]. An early retrospective cohort study in Hong Kong also found that patients treated with Paxlovid (*n =* 4,921) were at decreased risk of hospitalization, albeit with a much smaller risk reduction of 21% [5]. Another early study leveraging a large repository of Israeli health care data found a hazard ratio (HR) of 0.54 for severe COVID outcomes in patients treated with Paxlovid (*n =* 4,737) when compared with untreated patients, showing a protective association, but at a lower magnitude than the original EPIC-HR analysis [6]. A similar Israeli study, disaggregated by age, found a weaker protective association between Paxlovid and hospitalization within a cohort of patients aged 40 to 64 years (*n* = 66,433, HR = 0.74), and a stronger association for patients 65 years and over (*n* = 42,821, HR = 0.27) [7]. More recently, a study of 68,876 Cleveland Clinic patients with COVID-19 diagnoses from April 2022 to February 2023 found an adjusted hazard ratio of hospitalization of 0.63. That study was widely interpreted in news media as suggesting that Paxlovid may be less effective at preventing hospitalization caused by SARS-CoV-2 Omicron subvariants, although several earlier studies had similar findings [8,9]. To our knowledge, 2 studies have explicitly estimated Paxlovid’s causal effect on hospitalization in the real world. The first used a cohort of 44,551 patients from the Mass General Brigham health system in the northeastern US [10]. The second used a cohort of 256,288 patients from the US Department of Veterans Affairs (VA) database [11]. Both studies found a risk ratio of 0.60 for hospitalization. However, their study periods included the Omicron wave and ended in 2022, so the findings may not apply to current variants. The veteran population also differs from the general population in several relevant ways, including frequent history of traumatic brain injury, environmental exposures, and differing healthcare utilization patterns [12–16]. Together, these studies show a clear association between Paxlovid and reduced COVID-19 severity in real-world settings, but there remains a dearth of research specifically aimed at understanding the treatment effect of Paxlovid on COVID-19 outcomes with large, contemporary, national samples derived from real-world data.

Several EHR studies have also used real-world data to uncover racial and social disparities in the prescription of various COVID-19 treatments in the United States, including Paxlovid. Prior to Paxlovid’s authorization, multiple studies noted racial and social disparities among SARS-CoV-2–positive patients in access to and treatment with monoclonal antibodies (mAb), with black and Hispanic or Latino patients less likely than white patients to receive treatment with mAb [17–19]. A more recent large-scale study of EHR data revealed that those disparities have persisted with Paxlovid; from April through July of 2022, the rate of Paxlovid treatment was 35.8% lower among black adult patients than white adult patients [20].

Through the National Institute of Health’s (NIH) National COVID Cohort Collaborative (N3C), we expanded on prior studies of Paxlovid effectiveness and treatment patterns. We used the target trial emulation (TTE) framework and a large geographically and demographically diverse cohort from N3C’s EHR data repository [21]. Our study period of April 2022 through August 2023 provides up-to-date information to better assess Paxlovid’s effectiveness in the era of Omicron subvariant dominance. This study consisted of several analyses. First, we characterized the population prescribed Paxlovid and assessed potential disparities in Paxlovid prescription. Second, we estimated the causal effect of Paxlovid treatment on hospitalization and death among adults with COVID-19 in the US [21]. Third, building on prior work, we conducted a stratified analyses to estimate Paxlovid’s effect across vaccination status and age groups, that, to our knowledge, addresses an important gap in understanding the heterogeneity of its effectiveness across diverse patient populations.

## Methods

We performed a target trial emulation to assess the effect of Paxlovid treatment within 5 days of COVID-19 index on the risk of severe COVID-19 outcomes within 28 days of COVID-19 index. We followed a two-step process for emulating target trials with observational data: first, we articulated the causal question of interest in the form of a hypothetical randomized trial protocol, specifying eligibility criteria, treatment strategies, treatment assignment, the study period for follow-up, the outcome of interest, causal contrasts, and the analysis plan to estimate effects (Table 1) [22]. Second, we emulated each component of this protocol using patient-level data inside the NIH-hosted N3C Secure Data Enclave, which integrates EHR data for 18 million patients from 76 participating sites across the United States. N3C’s methods for patient data acquisition, ingestion, and harmonization have been reported in detail elsewhere [23–25]. All analyses and results as part of this study are reported in adherence with the Strengthening the Reporting of Observational Studies in Epidemiology (STROBE) reporting guidelines (S1 STROBE Checklist) [26].

**Table 1:** Protocol of a target trial to estimate the effect of paxlovid on the rate of hospitalization in the 28 days following a positive SARS-CoV-2 test.

Protocol component	Description under target trial conditions	Method of target trial emulation in this study
Eligibility criteria	Persons aged 18 years and older, who are not currently hospitalized and who have an acute COVID-19 infection, and are eligible for Paxlovid treatment due to presence of one or more risk factors for severe COVID-19 as per Center for US Centers for Disease and Prevention (CDC) guidelines [20].	Persons aged 18 years and older who are not currently hospitalized, who have one or more risk factors for severe COVID-19 as per CDC guidelines documented in their EHR and were not prescribed a drug with a severe interaction with Paxlovid in the 30 days prior to index, with a COVID-19 index (either a documented COVID-19 diagnosis or positive SARS-CoV-2 lab test) during the study period [27].
Treatment strategies	Paxlovid prescribed within a 5-day grace period of the date the patient presented with acute COVID-19.	Paxlovid prescribed within 5 days of COVID-19 index, indicated by a Paxlovid or nirmatrelvir drug exposure record in their EHR within 5 days of the COVID-19 diagnosis or positive SARS-CoV-2 lab test that constitutes their COVID-19 index date for the study.
Assignment procedures	Participants will be randomly assigned to treatment or control at the date they present with acute COVID-19 and will be aware of their treatment assignment.	Participants will be cloned across both treated and untreated arms, and will be assigned IPCW corresponding to their probability of remaining uncensored through the study period, to maintain the comparability of the study arms throughout the study period.
Follow-up period	Each patient will be followed for 28 days after treatment assignment. Patients who obtain Paxlovid from an outside source will be censored.	Patients will be censored at 28 days following treatment assignment across both groups post-cloning. Patients in the control group that deviate from their assigned treatment will be censored if they receive Paxlovid during the study period.
Outcome	Hospitalization within follow-up period	Hospitalization within follow-up period
Causal contrasts	Per-protocol effect	Per-protocol effect
Analysis plan	Estimation of effects via comparison of hospitalization rates among individuals assigned to each treatment arm, adjusting for pre-treatment covariates. Additional analyses to include the effects of vaccination and effect heterogeneity across age strata.	Estimation of cumulative incidence of hospitalization in each treatment arm using IPC-weighted Kaplan–Meier estimators; estimate the relative risk and absolute risk differences based on point estimates and variances of cumulative incidence estimates. Additional analyses to include the effects of vaccination, and effect heterogeneity across age strata.

COVID-19, Coronavirus Disease 2019; EHR, electronic health record; IPCW, inverse probability of censoring weights; SARS-CoV-2, Severe Acute Respiratory Syndrome Coronavirus 2.

### Eligibility criteria

We defined our study period as April 1, 2022 to August 28, 2023, with an index cutoff date of July 31, 2023. We excluded the period between December 21, 2021 (the date of Paxlovid’s EUA) and March 31, 2022, due to low treatment rates during this period and to make the study more relevant to the current phase of the pandemic. To meet the eligibility criteria for the study as per the target trial protocol, we specified the following inclusion criteria: (1) having a documented COVID-19 index date within the study period (with index date defined as the earliest date of either (a) COVID-19 diagnosis or (b) positive SARS-CoV-2 test result); (2) being ≥18 years of age as of the COVID-19 index date; (3) indicated for Paxlovid treatment by having one or more risk factors for severe COVID-19 as per CDC guidelines, including age ≥50 years old, or the presence of underlying medical conditions associated with a higher risk of severe COVID-19 [27]. We excluded all patients <18 years due to the potential for differences in both clinical characteristics and prescription practices among pediatric and adult patients [28,29].

We also specified 3 exclusion criteria: (1) patients who were hospitalized on or before the COVID-19 index date or date of treatment with Paxlovid (outcome precluding treatment); (2) patients who received Paxlovid before their COVID-19 index date; (3) patients who were prescribed a drug with a severe interaction with Paxlovid in the 30 days prior to the COVID-19 index [30]. In order to ensure that data were captured from sites with high fidelity and adequate coverage, we only included data from sites with at least 1% of eligible patients, and a minimum of 100 patients, treated with Paxlovid during the study period. This site-level exclusion criterion removed sites where Paxlovid was effectively unused, improving positivity across treatment arms.

### Treatment and outcome

Eligible patients were categorized by their treatment exposure, defined as receiving a Paxlovid prescription within a 5-day grace period of their COVID-19 index date, with controls defined as patients not prescribed Paxlovid in that period. We selected a 5-day grace period to parallel the clinical guideline to treat within 5 days of symptom onset and to minimize heterogeneity and potential for indication bias. Additionally, specifying a grace period and adjusting for this analytically allows us to avoid poorly defined interventions that may introduce errors in causal inference [31]. For patients who were never treated with Paxlovid, we used the date of their earliest indication of COVID-19 (diagnosis or positive lab result) within the study period as the index date. For those in the treatment group, we used their earliest indication of COVID-19 within 5 days of their first Paxlovid treatment date in the study period. Within the N3C enclave, the “Paxlovid or nirmatrelvir” concept set was used to identify drug exposures that correspond to Paxlovid (10 Observational Medical Outcomes Partnership [OMOP] concepts) [32]. We followed patients for a 28-day period following their COVID-19 index date. Our primary outcome was hospitalization at any point during the 28-day follow-up period. We also assessed death and a composite outcome of death or hospitalization as additional outcomes.

### Descriptive analysis

First, we applied two-sided Chi-squared tests to examine the distribution of Paxlovid treatment across 2 covariates: (1) patient race and ethnicity; and (2) a ZIP code-level community well-being index (hereafter referred to as CWBI). The CWBI measure is a composite index of social determinants of health available within the N3C database, with higher CWBI values corresponding to a higher level of protective community-level social determinants of health. The index methodology was developed by Sharecare and the Boston University School of Public Health. CWBI values are derived from the patient’s residential ZIP code-level data across 5 key inter-related community-level domains: healthcare access (ratios of healthcare providers to population), resource access (libraries and religious institutions, employment, and grocery stores), food access (access to grocery stores and produce), housing and transportation (home values, ratio of home value to income, and public transit use), and economic security (rates of employment, labor force participation, health insurance coverage rate, and household income above the poverty level) [33].

### Cloning, censoring, and weighting

Next, we used the potential outcomes framework to estimate the effect of Paxlovid treatment on hospitalization. We applied the clone-censor-weight technique, which is well-suited when the treatment grace period overlaps the outcome observation period [21,34]. This creates a period of “immortal time” for patients in the treatment group because they inherently survived without having the outcome until the time of treatment. This immortal time may bias estimates in favor of a lower risk in the treatment group, even in the absence of a true effect [31].

The clone-censor-weight technique assumes that, at time zero, all eligible patients could potentially be treated or not. We defined time zero (*t*_0_) as the date of a patient’s COVID-19 index date and *t*_A_ as the time of treatment assignment. For patients in the treatment group, *t*_A_ is the date of Paxlovid prescription within the 5-day grace period. For patients in the control group, *t*_A_ is day 5, when the grace period is over and treatment is no longer possible. We created a clone of each patient in our study cohort, with the clone assigned to the opposite treatment group of the true patient. Assigning each patient to both treatment strategies that are compatible with their observed data at *t*_0_ eliminates immortal time bias because the period between *t*_0_ and *t*_A_ counts as both treated and untreated [34,35]. It also makes the 2 treatment groups identical in pretreatment characteristics because they contain the same set of patients. This establishes exchangeability and removes confounding bias at *t*_0_.

Next, we applied artificial censoring to ensure that patients and clones follow their assigned treatment strategy after *t*_0_. Clones were censored at the time of their true patient counterpart’s *t*_A_. Therefore, clones of treated patients were censored from the control arm at the date of their true counterpart’s treatment. Clones of control patients were censored from the treated arm at day 5 when the grace period ended. In addition to this artificial censoring, for the hospitalization outcome, we treated death as a censoring event if it occurred posttreatment assignment, pre-outcome, within the study period.

Although the clone-censor-weight technique removes immortal time bias, it also introduces selection bias through informative artificial censoring [36]. To adjust for this selection bias, we applied inverse probability of censoring weights (IPCW) [37,38]. IPCW up-weights patients who remain at risk during the study period, such that the weighted cohort is equivalent to a cohort in which censoring is random. To calculate IPCW, we used logistic regression models to estimate the probability of remaining at risk at each day of the grace period, conditional on remaining at risk the previous day and on a set of covariates selected a priori based on clinical relevance to treatment and outcome. To convert daily probabilities to probabilities of remaining at risk through each day, we took the cumulative product of the daily probabilities. We stabilized the inverse of these probabilities and trimmed them to the 1st and 99th percentile. The value from day 5 (when all clones had been artificially censored) was carried forward through the rest of the study period.

IPCW covariates included sex, age (binned), race and ethnicity, prior history of individual comorbid conditions captured in the Charlson comorbidity index (CCI), value of the composite CCI (binned), prior history of conditions associated with risk of severe COVID-19 (as defined by the CDC Paxlovid eligibility criteria), CWBI (binned), number of visits in the year prior to index (binned), number of hospitalizations in the year prior to index (binned), month of COVID-19 onset, and site of care provision [27]. We included race, ethnicity, and CWBI as potential confounders due to the prevalence of literature suggesting disparity in treatment assignment and outcomes by these variables [17–20]. Sex, age, and comorbidities are known to affect both care-seeking and the outcome of COVID-19. The index month was included because Paxlovid treatment rates, viral variants, and infection rates changed during the study period. CCI was coded as missing when no condition exposures were present in N3C prior to index. CWBI was coded as missing when patient ZIP code was not reported.

### Effect estimation

We conducted an IPC-weighted analysis to estimate the effect of receiving Paxlovid (treatment), compared to not receiving Paxlovid (control), on the outcomes of hospitalization, death, and hospitalization, or death, among patients with acute COVID-19. Specifically, we estimated the cumulative incidence of each outcome in the 28 days following a COVID-19 infection using an IPC-weighted Kaplan–Meier estimator [38]. The weighted Kaplan–Meier estimator estimates the survival function as follows:

S^IPCWt =  1 if t < t1∏ti≤st1 -Σj:δjti = 1W^jtiΣk = 1nRktiW^ktiif t ≥ t1

*Where*:

- ${\hat{S}}_{IPCW}\left(t\right)\;$ is the weighted estimated survival function at time *t*.
- *t*_*i*_ are the event times.
- *δ*_*j*_(*t*_*i*_) is an indicator function (1 if individual *j* experiences the event at time *t*_*i*_, 0 otherwise).
- ${\hat{W}}_{j\left(t_{i}\right)}\;$ is the estimated weight for individual *j* at time *t*_*i*_.
- R_k_(t_i_) is an indicator function (1 if individual *k* is at risk at time *t*_*i*_, 0 otherwise).
- *n* is the total number of individuals.

We took the complement of the survival function as the cumulative incidence function. We estimated confidence intervals using a nonparametric bootstrap with 200 iterations. We present both absolute and relative risk estimates, and estimate number needed to treat (the number of patients who need to be treated to achieve 1 additional favorable outcome) as the reciprocal of the estimated absolute risk difference between treatment arms within the follow-up period [39–41].

### Vaccination-adjusted sub-analysis and stratified analysis

Our primary analysis did not consider COVID-19 vaccination status because vaccination information is subject to missingness in most EHRs. However, we conducted a vaccination-sub-analysis in a subset of sites with high-quality vaccination data. We used the same method applied to the primary analysis described above with an additional indicator of whether or not the patient was fully vaccinated at least 2 weeks prior to index included as a covariate. We considered this important for 2 reasons. First, we hypothesized that vaccination status may be a confounder of Paxlovid treatment and hospitalization, largely through the latent variable of infection severity or propensity to seek care. If so, our primary analysis would violate the assumption of no unmeasured confounding. Second, we hypothesized that Paxlovid treatment may be less effective among vaccinated patients. There is mixed evidence for this dynamic. Pfizer’s Evaluation of Protease Inhibition for COVID-19 in Standard-Risk Patients (EPIC-SR) trial found no significant treatment effect among vaccinated patients with one risk factor for severe COVID-19 [42,43]. A large health system found that Paxlovid treatment reduced the likelihood of hospitalization and death among vaccinated patients, but that the treatment effect was smaller than among unvaccinated patients [10].

For this sub-analysis, we used a modified cohort of patients from sites with reliable information on patient vaccination status. Vaccination status in N3C is subject to misclassification, particularly among patients who were vaccinated outside of data partner systems. We determined the subset of sites with reliable patient-level vaccination records by the ratio of 2 statistics: (1) the proportion of individuals who are documented as vaccinated in their EHR; and (2) the proportion of individuals who are truly vaccinated. We calculated the first statistic from the EHR data for each site. We estimated the second statistic using CDC-reported vaccination rates for the counties served by each partner facility [44]. CDC vaccination rates by county are included as a data asset in N3C. Patient counties were inferred using a county-ZIP crosswalk. Each patient’s likelihood of vaccination was drawn from their county’s vaccination rate and the overall expected vaccination rate for a partner facility was computed as the mean of their patients’ vaccination likelihood. We defined a facility’s recorded vaccine ratio as the ratio of these 2 statistics and limited the vaccination sub-analysis to individuals from facilities with a ratio of at least 0.66 [45].

Accordingly, we categorized patients by their vaccination status prior to their COVID-19 index date, defined as having completed a full course of vaccination at least 14 days prior to index. Partially vaccinated patients and patients who were fully vaccinated fewer than 14 days prior to index were excluded from the analysis. We estimated the absolute and relative treatment effect of Paxlovid on hospitalization in this cohort using the same statistical analysis steps described for the primary analysis.

We also conducted a stratified analysis to examine effect heterogeneity by vaccination status. For fully vaccinated and unvaccinated patients in the vaccination-aware cohort, we separately estimated the absolute and relative treatment effect of Paxlovid on hospitalization using the same statistical analysis steps described for the primary analysis.

### Age-stratified analysis

We conducted a second stratified analysis to examine effect heterogeneity across 3 strata of patient age at COVID-19 index: 18 to 49 years, 50 to 64 years, and 65+ years. We selected these strata due to their clinical and policy implications. The first cutoff, age 50 years, was grounded in the eligibility criteria for treatment with Paxlovid. Patients below 50 years old are not indicated for treatment unless they have additional risk factors for severe COVID-19. We chose the second cutoff of age of 65 years due to its policy relevance as an eligibility cutoff for health coverage through Medicare, and consequent increase in utilization of services [46].

### Ethics approval

The N3C data transfer to the National Center for Advancing Translational Sciences is performed under a Johns Hopkins University reliance protocol (IRB00249128). The N3C Publications Committee approved this manuscript (manuscript ID 1020.56). Results downloads were approved per N3C Attribution and Publication Principles [47].

## Results

**Fig 1:**
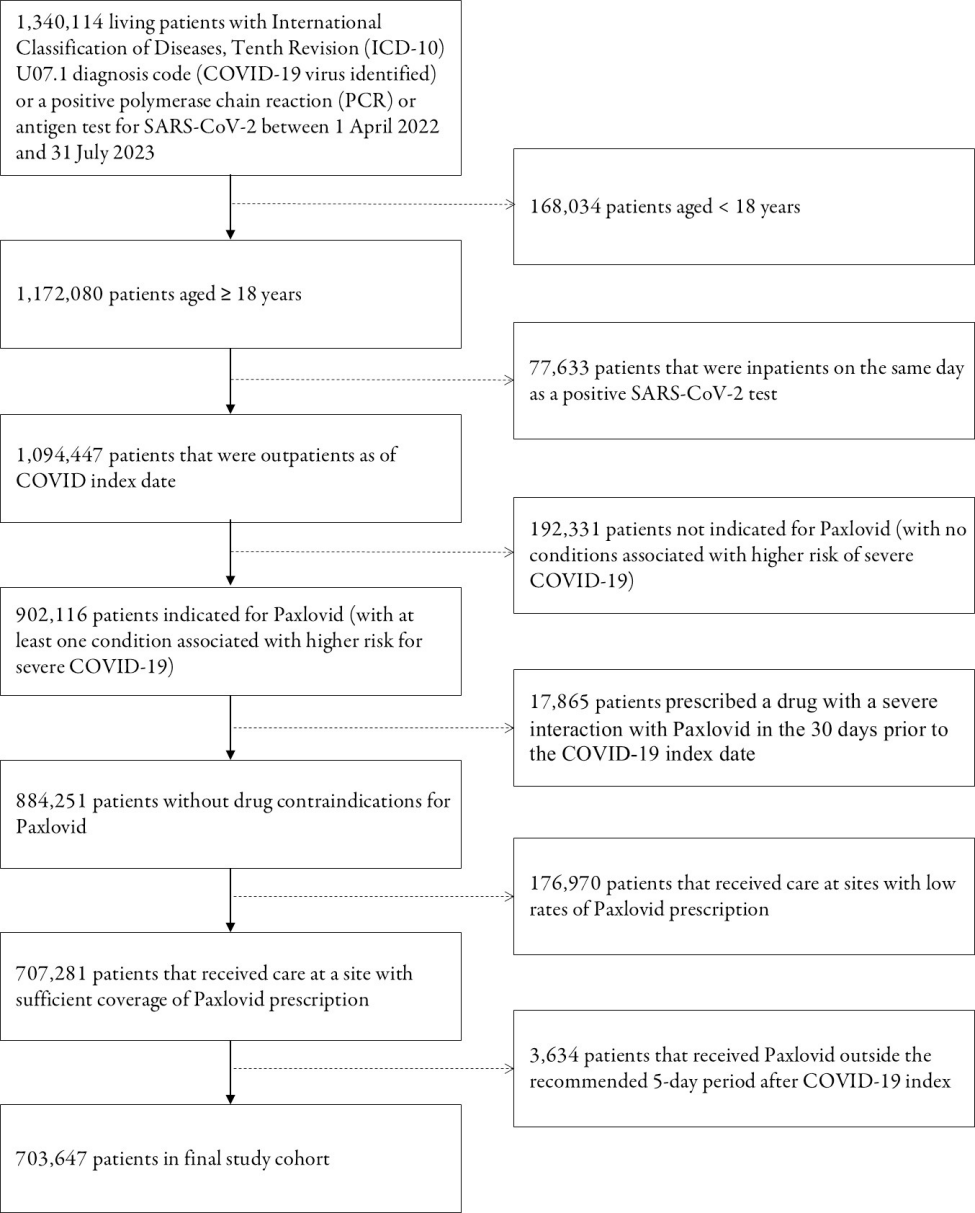
Study cohort and flow of emulated trial. COVID-19, Coronavirus Disease 2019; SARS-CoV-2, Severe Acute Respiratory Syndrome Coronavirus 2.

The hypothesized target trial protocol is articulated in Table 1, and we emulated each component to define our cohort within the N3C database (see Fig 1).

### Patient characteristics

In the study cohort (within our defined study period), a total of 703,647 patients had a valid COVID-19 index date during the study period, of which 206,393 (20.6%) were treated with Paxlovid, and a total of 13,895 (2.0%) patients across the entire study cohort were hospitalized. After applying the eligibility criteria to the patient population and selecting high-fidelity Paxlovid prescription sites, a total of 34 of 76 study sites were retained. The characteristics of all patients during the study period are presented in Table 2, stratified by treatment group.

**Fig 2:**
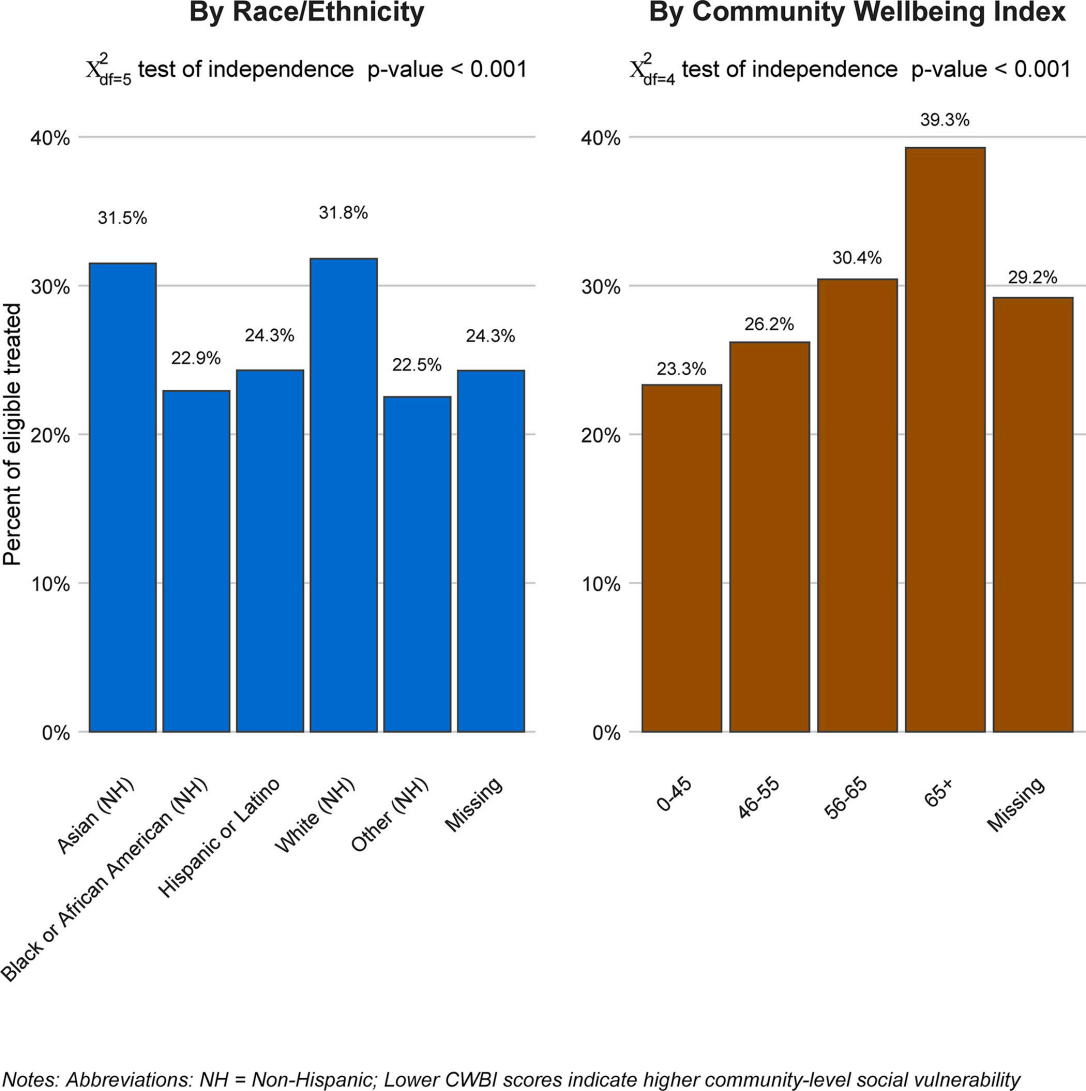
Paxlovid treatment rates by patient race and ethnicity and CWBI. CWBI, community well-being index.

Among the study cohort, there were large, statistically significant differences in assignment to Paxlovid treatment. In our study cohort, 15.7% of black, non-Hispanic patients and 15.1% of Hispanic patients were treated with Paxlovid, compared to 22.4% of white, non-Hispanic patients, and 23.1% of Asian, non-Hispanic patients (*X*^2^_df = 5_ test of independence *p*-value <0.001). When stratified by patients’ residential areas, patients who lived in areas with higher CWBI values (and lower corresponding social vulnerability) were also more likely to be treated with Paxlovid (*X*^2^_df = 4_ test of independence *p*-value <0.001; see Fig 2). The relationship between race and ethnicity, and CWBI values across the study cohort of included patients is provided in S1 Fig.

**Table 2:** Baseline population characteristics.

Characteristic	Treatment group	
	No Paxlovid	Paxlovid
	(*N* = 497,085)	(*N* = 206,562)
Hospitalization (within 28-day observation period)		
No	485,091 (97.6%)	204,661 (99.1%)
Yes	11,994 (2.4%)	1,901 (0.9%)
Sex		
Female	312,104 (62.8%)	127,854 (61.9%)
Male	184,903 (37.2%)	78,670 (38.1%)
Missing	78 (0.0%)	38 (0.0%)
Age (in years)		
18–24	30,332 (6.1%)	4,722 (2.3%)
25–34	65,971 (13.3%)	14,825 (7.2%)
35–49	102,845 (20.7%)	37,479 (18.1%)
50–64	141,569 (28.5%)	66,275 (32.1%)
65+	156,368 (31.5%)	83,261 (40.3%)
Race and ethnicity		
Asian non-Hispanic	20,391 (4.1%)	9,374 (4.5%)
Black or African American non-Hispanic	68,292 (13.7%)	20,301 (9.8%)
Hispanic or Latino any Race	49,826 (10.0%)	16,000 (7.7%)
White non-Hispanic	316,839 (63.7%)	147,751 (71.5%)
Other non-Hispanic	8,123 (1.6%)	2,359 (1.1%)
Unknown	33,614 (6.8%)	10,777 (5.2%)
CCI2		
0	249,618 (50.2%)	101,444 (49.1%)
1–2	132,010 (26.6%)	66,593 (32.2%)
3–4	42,029 (8.5%)	18,421 (8.9%)
5–10	25,557 (5.1%)	8,821 (4.3%)
11+	2,798 (0.6%)	820 (0.4%)
Missing	45,073 (9.1%)	10,463 (5.1%)
Number of clinical visits in prior year		
0	70,533 (14.2%)	21,843 (10.6%)
1–3	84,908 (17.1%)	23,641 (11.4%)
4–9	102,173 (20.6%)	41,641 (20.2%)
10–20	107,292 (21.6%)	54,840 (26.5%)
More than 20	132,179 (26.6%)	64,597 (31.3%)
Number of hospitalizations in prior year		
0	465,424 (93.6%)	197,380 (95.6%)
1 visit	23,513 (4.7%)	7,328 (3.5%)
More than 1 visit	8,148 (1.6%)	1,854 (0.9%)
CWBI3		
0–45	4,082 (0.8%)	1,241 (0.6%)
46–55	185,995 (37.4%)	65,984 (31.9%)
56–65	223,789 (45.0%)	97,878 (47.4%)
65+	30,591 (6.2%)	19,777 (9.6%)
Missing	52,628 (10.6%)	21,682 (10.5%)
SARS-CoV-2 test type		
Antibody	498 (0.1%)	38 (0.1%)
Antigen	71,846 (16.0%)	18,318 (17.9%)
Molecular (polymerase chain reaction and nucleic acid amplification tests)	424,199 (79.2%)	54,623 (77.9%)
Other	27,910 (4.7%)	1,877 (2.3%)
Deaths within 28-day observation period		
No	495,773 (99.7%)	206,435 (99.9%)
Yes	1,312 (0.3%)	127 (0.1%)
Intermittent mandatory ventilation during hospitalization		
No	496,810 (99.9%)	206,511 (100.0%)
Yes	275 (0.1%)	51 (0.0%)
Month of COVID-19 diagnosis		
Apr 2022	25,584 (5.1%)	5,088 (2.5%)
May 2022	60,027 (12.1%)	17,910 (8.7%)
Jun 2022	62,628 (12.6%)	20,808 (10.1%)
Jul 2022	70,240 (14.1%)	26,357 (12.8%)
Aug 2022	58,862 (11.8%)	21,618 (10.5%)
Sep 2022	38,479 (7.7%)	14,827 (7.2%)
Oct 2022	29,224 (5.9%)	11,297 (5.5%)
Nov 2022	27,750 (5.6%)	12,895 (6.2%)
Dec 2022	38,098 (7.7%)	22,070 (10.7%)
Jan 2023	28,537 (5.7%)	15,140 (7.3%)
Feb 2023	18,970 (3.8%)	12,044 (5.8%)
Mar 2023	14,148 (2.8%)	9,392 (4.5%)
Apr 2023	8,733 (1.8%)	5,879 (2.8%)
May 2023	6,651 (1.3%)	4,632 (2.2%)
Jun 2023	4,758 (1.0%)	3,251 (1.6%)
Jul 2023	4,396 (0.9%)	3,354 (1.6%)

^1^Any hospital admission in the 28-days following a positive SARS-CoV-2 test result.

^2^CCI is a weighted index to predict risk of death within 1 year of hospitalization for patients with specific comorbid conditions, and this implementation uses the weights from Quan and colleagues [48,49].

^3^CWBI is a measure of 5 interrelated community-level domains: Healthcare access (ratios of healthcare providers to population), resource access (libraries and religious institutions, employment, and grocery stores), food access (access to grocery stores and produce), housing and transportation (home values, ratio of home value to income, and public transit use), and economic security (rates of employment, labor force participation, health insurance coverage rate, and household income above the poverty level) [33].

CCI, Charlson comorbidity index; CWBI, community well-being index; SARS-CoV-2, Severe Acute Respiratory Syndrome Coronavirus 2.

### Effect of Paxlovid on hospitalization

**Fig 3:**
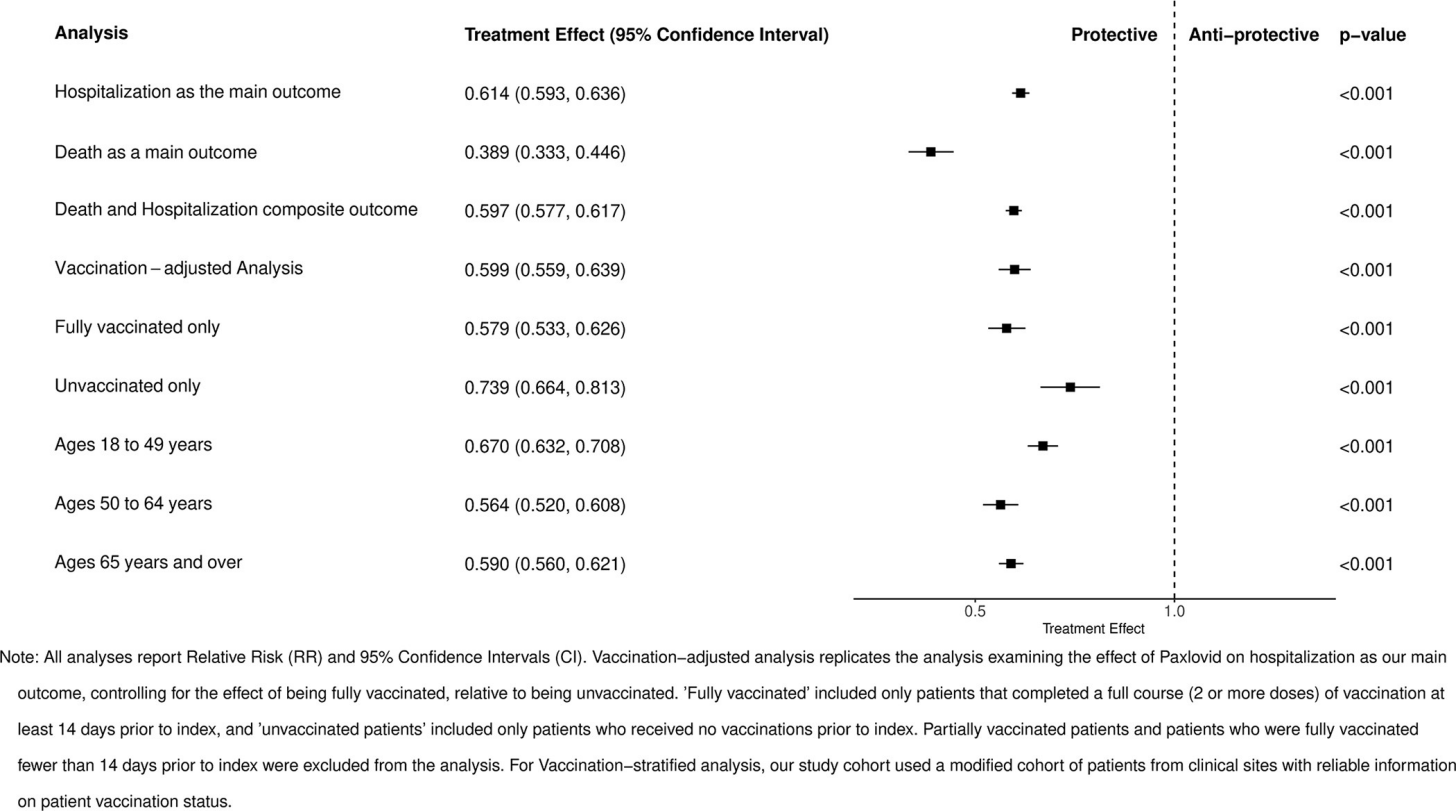
Forest plot of treatment effects of Paxlovid on patients with COVID-19 across all analyses (reported as relative risks). CI, confidence interval; COVID-19, Coronavirus Disease 2019; RR, relative risk.

In our study cohort, hospitalization rates differed markedly between Paxlovid-treated and untreated patients. Among those treated with Paxlovid, 1,901 (0.9%) were hospitalized during the follow-up period, compared to 11,994 (2.4%) in the untreated group. Our primary analysis, utilizing a cloned, censored, and IPC-weighted cohort, revealed that Paxlovid treatment was associated with a significantly lower relative risk (RR, 0.614; 95% CI [0.593, 0.636]; *p* ≤ 0.001). Additionally, the estimated absolute risk difference (ARD) indicated a substantive absolute risk reduction in the treated cohort (ARD, 0.009; 95% CI [0.009, 0.010]; *p* ≤ 0.001), corresponding to a number needed to treat (NNT) of 111 to avert 1 hospitalization during the follow-up period (see Fig 3 and Table 3).

**Table 3:** Estimated cumulative incidence, RRs, absolute risk reduction, and numbers needed to treat associated with treatment with Paxlovid across all analyses.

Analysis	Cumulative incidence (95% CI)		RR (95% CI)	Absolute risk difference (95% CI)	NNT2
	Paxlovid	No Paxlovid			
**Main results**					
Hospitalization as the main outcome	0.015 (0.014, 0.015)	0.024 (0.023, 0.024)	0.614 (0.593, 0.636)*	0.009 (0.009, 0.010)*	111 (100, 111)
Death as a main outcome	0.001 (0.001, 0.001)	0.003 (0.003, 0.003)	0.389 (0.333, 0.446)*	0.002 (0.001, 0.002)*	500 (500, 1,000)
Death and hospitalization composite outcome	0.015 (0.015, 0.016)	0.026 (0.025, 0.026)	0.597 (0.577, 0.617)*	0.010 (0.010, 0.011)*	100 (91, 100)
**Vaccination, stratified analysis**1					
Vaccination-adjusted analysis	0.011 (0.010, 0.011)	0.018 (0.018, 0.019)	0.599 (0.559, 0.639)*	0.007 (0.006, 0.008)*	143 (125, 167)
Fully vaccinated patients only	0.009 (0.009, 0.010)	0.016 (0.016, 0.017)	0.579 (0.533, 0.626)*	0.007 (0.006, 0.008)*	143 (125, 167)
Unvaccinated patients only	0.017 (0.016, 0.019)	0.023 (0.022, 0.025)	0.739 (0.664, 0.813)*	0.006 (0.004, 0.008)*	167 (125, 250)
**Age, stratified analysis**					
Ages 18 to 49 years	0.015 (0.014, 0.016)	0.022 (0.021, 0.023)	0.670 (0.632, 0.708)*	0.007 (0.006, 0.008)*	143 (125, 167)
Ages 50 to 65 years	0.009 (0.009, 0.010)	0.016 (0.016, 0.017)	0.564 (0.520, 0.608)*	0.007 (0.006, 0.008)*	143 (125, 167)
Ages 65+ years	0.019 (0.018, 0.020)	0.033 (0.032, 0.033)	0.590 (0.560, 0.621)*	0.013 (0.012, 0.015)*	77 (67, 84)

*p < 0.001.

^1^Vaccination-adjusted analysis replicates the analysis examining the effect of Paxlovid on hospitalization as our main outcome, controlling for the effect of being fully vaccinated, relative to being unvaccinated. “Fully vaccinated” included only patients that completed a full course (2 or more doses) of vaccination at least 14 days prior to index, and “unvaccinated patients” included only patients who received no vaccinations prior to index. Partially vaccinated patients and patients who were fully vaccinated fewer than 14 days prior to index were excluded from the analysis. For Vaccination-stratified analysis, our study cohort used a modified cohort of patients from clinical sites with reliable information on patient vaccination status.

^2^NNT calculated as (1 ÷ absolute risk difference), corresponding to the expected number of patients that must be treated to prevent the outcome in one patient within the specified follow-up period, rounded up to the nearest integer.

CI, confidence interval; COVID-19, Coronavirus Disease 2019; RR, relative risk.

### Effect of Paxlovid on death and composite outcome

We also evaluated the effect of Paxlovid on mortality and a composite outcome of hospitalization and death (see Fig 3 and Table 3). Among Paxlovid-treated patients, 127 (0.1%) died during follow-up, compared to 1,312 (0.3%) in the untreated group. Paxlovid treatment was associated with a significantly lower relative risk of death (RR, 0.389; 95% CI [0.333, 0.446]; *p* ≤ 0.001) and a small but meaningful absolute risk reduction (ARD, 0.002; 95% CI [0.001, 0.002]; *p* ≤ 0.001), corresponding to an NNT of 500 to avert 1 death during the follow-up period. For the composite outcome of hospitalization and death, Paxlovid treatment similarly showed both a lower relative risk (RR, 0.597; 95% CI [0.577, 0.617]; *p* ≤ 0.001) and a significant absolute risk reduction (ARD, 0.010; 95% CI [0.010, 0.011]; *p* ≤ 0.001), corresponding to an NNT of 100 to avert 1 hospitalization or death during the follow-up period. These results demonstrate that Paxlovid not only reduces the risk of hospitalization but also significantly lowers mortality risk, with the composite outcome reinforcing its overall clinical benefit.

### Effect of Paxlovid by COVID-19 vaccination status

For our subgroup analysis estimating the effect of Paxlovid on hospitalization, we examined both vaccination status and age groups (see Fig 3 and Table 3). The vaccination analysis included 272,080 vaccinated individuals (29.1% received Paxlovid, 1.3% hospitalized) and 106,832 unvaccinated individuals (14.1% received Paxlovid, 2.3% hospitalized) from 9 sites with trusted vaccine data. After cloning, censoring, and adjusting for vaccination status, the overall relative risk (RR, 0.599; 95% CI [0.559, 0.639]; *p* ≤ 0.001) and absolute risk reduction (ARD, 0.007; 95% CI [0.006, 0.008]; *p* ≤ 0.001) were similar to the primary analysis, with an estimated NNT of 143 to avert 1 hospitalization during the follow-up period. Fully vaccinated and unvaccinated patients showed similar absolute risk reductions with Paxlovid (ARD, 0.007 versus 0.006), but vaccinated patients had a larger relative risk reduction (RR, 0.579 versus 0.739) due to their lower baseline hospitalization risk.

### Effect of Paxlovid by age group

In our age-stratified analysis, we observed varying effects of Paxlovid across different age groups (see Fig 3 and Table 3). Notably, while Paxlovid provides a benefit across all age groups, the absolute risk reduction is most pronounced in older adults, likely due to their higher baseline risk of hospitalization. For individuals aged 18 to 49 years, Paxlovid treatment was associated with a relative risk of 0.670 (95% CI [0.633, 0.708]; *p* ≤ 0.001) and an absolute risk reduction of 0.007 (95% CI [0.006, 0.008]; *p* ≤ 0.001) for hospitalization, corresponding to an NNT of 143 to avert 1 hospitalization during the follow-up period. In the 50 to 65 years age group, the relative risk was 0.564 (95% CI [0.520, 0.608]; *p* ≤ 0.001) with an absolute risk reduction of 0.007 (95% CI [0.006, 0.008]; *p* ≤ 0.001), also corresponding to an NNT of 143 to avert 1 hospitalization during the follow-up period. The greatest benefit was observed in the 65+ years age group, where Paxlovid treatment resulted in a relative risk of 0.590 (95% CI [0.560, 0.621]; *p* ≤ 0.001) and an absolute risk reduction of 0.013 (95% CI [0.012, 0.015]; *p* ≤ 0.001), corresponding to an NNT of 77 to avert 1 hospitalization during the follow-up period.

## Discussion

In this target trial emulation using the N3C database, Paxlovid treatment within 5 days of a COVID-19 diagnosis or positive SARS-CoV-2 test reduced the risk of hospitalization by 39%, death by 61%, and hospitalization or death by 40%. The effect size for hospitalization is similar to the association found in a Cleveland Clinic study (37%), which also used a contemporary study period, and the effect size found in VA and Mass General Brigham studies (40%), which also used target trial emulation methods [9–11]. We confirm these findings using a larger, more representative cohort. Although other studies have found varying effect sizes, we believe this constitutes a growing consensus on the magnitude of Paxlovid’s effect in the real world. While our findings are consistent with other real-world studies, they differ from the results of early clinical trials like EPIC-HR. This discrepancy is likely due to differences in study design rather than changes in virus variants. Factors such as inclusion criteria, timing of treatment initiation, and estimation of intention-to-treat versus per-protocol effects may account for the lower effectiveness observed in real-world settings compared to clinical trials.

The Cleveland Clinic study was widely interpreted in the media with headlines like “Paxlovid Weaker Against Current COVID-19 Variants” and “Paxlovid Now Less Effective Than In Early Trials” [8,9]. We consider that to be a misinterpretation. Earlier real-world studies found similar or lower effect sizes in study periods dominated by earlier variants [5,10,11]. Real-world studies of Paxlovid’s effectiveness have also differed in population, sampling, outcomes, and methodology, and these are more likely than the dominant variant during the study period to account for their differences. It is true that real-world studies have consistently found a lower risk reduction than in the EPIC-HR trial. This may be attributable to various differences in study design (as noted by Najjar-Debbiny and colleagues): (1) differences in dominant strains at the time of the study; (2) inclusion of only symptomatic patients versus inclusion of all COVID-19 positive patients (as well as other possible cohort or methodological differences); (3) treatment assignment within 5 days of symptom onset versus treatment assignment within 5 days of a COVID-19 diagnosis or a positive SARS-CoV-2 test; and (4) estimation of the intention-to-treat effect versus the per-protocol effect [6]. We consider the latter to be the most likely hypothesis, but further research on the source of this difference is needed. Regardless, Paxlovid is not less effective now than in early trials, it is less effective in the real world than in early trials. This common misinterpretation illustrates how media reports often draw the wrong conclusions from scientific research, and why the scientific community must strive to interpret findings for lay audiences.

In a subcohort with reliable vaccination data, we found that Paxlovid treatment reduced the absolute risk of hospitalization by 0.7 percentage points, regardless of vaccination status. This reduction in absolute risk translates to an NNT of 143, meaning that for every 143 individuals treated with Paxlovid, one hospitalization would be prevented. While the relative risk reduction (RRR) for vaccinated patients was larger (42% versus 26% for unvaccinated patients), the consistent ARD across both groups suggests that the benefit of Paxlovid in preventing hospitalization is similar for both vaccinated and unvaccinated populations. Although the NNT is high, particularly in vaccinated patients with lower baseline risk, it is important to contextualize this within the broader public health system. In high-risk populations, even a modest reduction in absolute risk can have significant impacts, particularly in reducing the strain on the healthcare system. Additionally, the consistency of the absolute risk reduction regardless of vaccination status highlights the additive nature of the protective effects of vaccination and Paxlovid, rather than a multiplicative effect. This supports the use of Paxlovid as a valuable treatment option for preventing severe COVID-19 outcomes, even among vaccinated patients.

This is particularly noteworthy given the preliminary evidence from the EPIC-SR trial and other real-world studies that suggested uncertainty about Paxlovid’s impact on vaccinated populations [10,42,43]. Our results indicate that Paxlovid treatment remains valuable even for vaccinated patients, providing a consistent absolute risk reduction regardless of vaccination status. Further research is needed on the effectiveness and cost-effectiveness of Paxlovid treatment in vaccinated populations. It is also worth noting that the overall effect of Paxlovid treatment in this subcohort while controlling for vaccination status was similar to the primary analysis (40% relative risk reduction of hospitalization). This suggests that vaccination status did not cause substantial unmeasured confounding in the primary analysis.

In an age-stratified analysis, we found a larger absolute risk reduction for patients aged 65+ years (1.3 percentage points) compared to patients ages 18 to 49 years and 50 to 64 years (0.7 percentage points for both groups). This ARD translates into an NNT of 77 for patients aged 65+, implying that fewer individuals in this age group need to be treated to avert one hospitalization. Overall hospitalization risk was highest among patients aged 65+ years, followed by patients ages 18 to 49 years. Higher overall risk among patients ages 18 to 49 than among patients ages 50 to 64 years is explained by the cohort inclusion criteria. All patients over 50 years are indicated for Paxlovid treatment, and therefore eligible for inclusion, while patients under 50 years require a specific comorbidity associated with increased risk of severe COVID-19 to be indicated for treatment. This likely results in a higher-risk subcohort in this age group. Due to these differences in overall risk, the 18 to 49 years age group had the lowest RRR (33%), followed by the 65+ years group (41%) and the 50 to 64 years group (44%). Our findings suggest that older patients may have the most to gain from Paxlovid treatment, but further research is needed to understand the potential population health impact of differing treatment protocols by age group.

We also found large differences in Paxlovid treatment rates by race, ethnicity, and CWBI. Paxlovid is thus effective at reducing the risk of severe COVID-19 outcomes but is not equitably distributed. Black and Hispanic/Latino patients were less likely to receive Paxlovid treatment than white and Asian patients. Additionally, patients from communities with higher levels of social vulnerability (lower CWBI values) were less likely to receive Paxlovid treatment. Prior research from December 2021 to July 2022 documented similar disparities [20]. Our findings show that these disparities have persisted over time. These disparities in access to treatment are of particular concern due to the growing evidence base that shows that individuals in these groups are more likely to experience higher levels of COVID-19 exposure, discrimination in access to care, and severe COVID-19 health outcomes [50–55]. The reasons for these disparities are a complex constellation of factors including (and not limited to) a history of systemic discrimination and racism in treatment, lack of physical and economic resources to facilitate equal access in vulnerable communities, lack of information and knowledge about treatment options, and language barriers owing to a lack of culturally competent care [20]. Without attention, recognition, and remediation on the part of providers, public health agencies, the health system, and communities, the disproportional burden of COVID-19 will only further exacerbate existing health inequities in the US.

This study has several strengths that underscore the value of large-scale EHR repositories for advancing comparative effectiveness research. In the absence of large-scale randomized controlled trials, target trial emulations allow researchers to explore treatment effects in real-world settings and identify the most effective treatments for a variety of health conditions. This study is one of a few studies that apply methods to emulate hypothetical target trials, accounting for the effect of confounding [56–59]. Additionally, the analyses were conducted using a large, comprehensive database of EHR data from 34 contributing sites across the US, increasing generalizability, and decreasing the potential for issues that typically arise from misclassification in administrative or claims data [60]. We used a contemporary study period, which makes our findings more relevant to current SARS-CoV-2 subvariants than many prior studies on the topic. Finally, to our knowledge, our stratified analyses contribute novel findings on Paxlovid’s heterogeneous effects by age group and homogeneous effects by vaccination status, providing valuable insights for targeted treatment strategies.

This study also has several limitations. First, since the data did not include any information on adherence (Paxlovid is typically given orally twice daily for 5 days), we were unable to account for adherence. Second, the sub-analysis on vaccinations did not include individuals with incomplete courses of vaccination (1 dose), nor information on the timing of vaccination relative to COVID-19 infection, and therefore we were unable to shed light on whether the response to Paxlovid varied by additional strata of vaccination. Third, it is well-documented that EHRs are susceptible to missing data when patients do not seek care, care is provided outside of the reporting facility, or a condition is documented outside of the structured EHR (e.g., in clinical notes), and it is likely that our estimates may be biased if missingness was related to any residual unobserved confounding [61–63]. We are also unable to assess the reason for hospitalization, as N3C does not have information on the priority ranking of discharge diagnoses. We took several steps to mitigate the risk of missing data—all individuals in our cohort have established care at the partner facility before their COVID-19 index, as evidenced by documented healthcare encounters, and our vaccination sub-analysis is limited to facilities with a high recorded vaccine ratio to reduce the number of individuals misclassified as unvaccinated. Related to this inclusion criteria, there may have been site-level variation in Paxlovid treatment rates across sites, such as differences in institutional protocols, local COVID-19 prevalence, patient demographics, and healthcare system capacity—while we may not have been able to fully account for these factors within the scope of our analysis, future work may benefit from addressing factors that may introduce site-level confounding in treatment assignment. Fourth, our inclusion criteria of Paxlovid treatment within 5 days of COVID-19 index differs from the indication of treatment within 5 days of symptom onset. However, we note that within our base cohort, 85.1% of treated patients were treated within 1 day of COVID-19 index. Fifth, this study’s eligibility criteria include indication for on-label Paxlovid treatment (i.e., at risk for developing severe COVID-19 due to the presence of 1 or more risk factors). Therefore, results can only be generalized to a high-risk population. The effect of Paxlovid treatment among lower-risk patients is an important area for future research, although studying it in the real world is difficult due to confounding by indication. Finally, our study is subject to the assumptions required of all causal inference studies: consistency, positivity, and exchangeability. In particular, the assumption of exchangeability rests on the assumption that there are no unmeasured confounders. Although we controlled for a wide range of potential confounders, certain possible confounders were unmeasurable in the EHR. Patient insurance status and economic well-being could affect decisions to seek Paxlovid and hospitalization. Provider behavior related to offering Paxlovid treatment also varies in this cohort, as shown by the variation in treatment rates by site. Treatment availability may also contribute to that variation. Ultimately, although we are confident that we controlled for confounding to the greatest extent possible, residual unmeasured confounding is possible.

We found that Paxlovid treatment within 5 days of a COVID-19 diagnosis or positive test reduced the risk of hospitalization by 39%. Broadly speaking, our findings are consistent with the evidence base: Paxlovid is effective at preventing severe COVID-19 outcomes, but it is less effective in real-world settings than in early clinical trials. We used a study period (April 2022 to August 2023) that included COVID-19 cases caused mostly by SARS-CoV-2 Omicron subvariants, which suggests that Paxlovid remains effective against more recent variants. We found that Paxlovid treatment reduced the absolute risk of hospitalization by the same amount (0.7 percentage points), regardless of vaccination status, which suggests that Paxlovid treatment is valuable even for vaccinated patients. We also found that Paxlovid had the greatest absolute risk reduction among patients aged 65+ years (1.3 percentage points), compared to patients ages 18 to 49 years and 50 to 64 years (0.7 percentage points for both groups), which suggests that older patients may have the most to gain from Paxlovid treatment. Finally, we found disparities in the rates of Paxlovid treatment. Black patients, Hispanic or Latino patients, and patients living in more vulnerable communities were treated with Paxlovid at a significantly lower rate than others. Taking action to remediate these disparities will equalize the opportunity for all high-risk patients to prevent severe COVID-19 outcomes.

## Ethics approval and consent to participate

The N3C data transfer to NCATS is performed under a Johns Hopkins University Reliance Protocol #IRB00249128 or individual site agreements with NIH. The N3C Data Enclave is managed under the authority of the NIH; information can be found at https://ncats.nih.gov/n3c/resources. The work was performed under DUR RP-5677B5.

## Supporting information

S1 STROBE Checklist STROBE Statement—Checklist of items that should be included in reports of cohort studies. (DOCX)

S1 Fig Proportion of individuals in base population stratified by race and ethnicity, and ZIP code-level community wellbeing index (CWBI). (DOCX)

## Abbreviations

ARD: absolute risk difference
CCI: Charlson comorbidity index
CI: confidence interval
COVID-19: Coronavirus Disease 2019
CWBI: community well-being index
EHR: electronic health record
EUA: Emergency Use Authorization
FDA: Food and Drug Administration
HR: hazard ratio
IPCW: inverse probability of censoring weights
mAb: monoclonal antibodies
N3C: National COVID Cohort Collaborative
NNT: number needed to treat
NIH: National Institute of Health
RR: relative risk
RRR: relative risk reduction
SARS-CoV-2: Severe Acute Respiratory Syndrome Coronavirus 2
TTE: target trial emulation
VA: Veterans Affairs
