# Supplementary material for: Effect of nirmatrelvir/ritonavir (Paxlovid) on hospitalization among adults with COVID-19: An electronic health record-based target trial emulation from N3C
Source: PLoS Med. 2025 Jan 17;22(1):e1004493. doi: 10.1371/journal.pmed.1004493 (PMC11790232; doi:10.1371/journal.pmed.1004493)
Supplement: S1 STROBE Checklist — (DOCX) [file pmed.1004493.s001.docx]

**S1 STROBE Checklist-** STROBE Statement—Checklist of items that should be included in reports of cohort studies.

|  | Item No. | Recommendation | Section | Relevant text from manuscript |
| --- | --- | --- | --- | --- |
| **Title and abstract** | 1 | (*a*) Indicate the study’s design with a commonly used term in the title or the abstract | Abstract – Background, Paragraph 1 | This study leverages electronic health record data in the National COVID Cohort Collaborative's (N3C) repository to investigate disparities in Paxlovid treatment and to emulate a target trial assessing its effectiveness in reducing severe COVID-19 outcomes. |
|  |  | (*b*) Provide in the abstract an informative and balanced summary of what was done and what was found | Abstract – Methods and Findings, Paragraph 2 | In a cohort of 703,647 COVID-19 patients seen at 34 clinical sites across the United States between April 1, 2022 and August 28, 2023 … we estimated that Paxlovid reduced the risk of hospitalization by 39% (95% confidence interval [CI] 36, 41%), with an absolute risk reduction of 0.9 percentage points (95% CI 0.9, 1.0). We also found that Paxlovid reduced the risk of death by 61%, and the risk of hospitalization or death by 40%. |
| Introduction | | | |  |
| Background/rationale | 2 | Explain the scientific background and rationale for the investigation being reported | Introduction, Paragraph 1 | In December 2021, the US Food and Drug Administration (FDA) issued an Emergency Use Authorization (EUA) for Paxlovid, enabling its prescription to high-risk SARS-CoV-2-positive patients aged 12 and older… but there remains a dearth of research specifically aimed at understanding the treatment effect of Paxlovid on COVID-19 outcomes with large, contemporary, national samples derived from real-world data. |
| Objectives | 3 | State specific objectives, including any prespecified hypotheses | Introduction, Paragraph 4 | First, we characterize the population prescribed Paxlovid and assess potential disparities in Paxlovid prescription. Second, we estimate the causal effect of Paxlovid treatment on hospitalization and death among adults with COVID-19 in the United States. |
| Methods | | | |  |
| Study design | 4 | Present key elements of study design early in the paper | Methods, Paragraph 1 | We performed a target trial emulation to assess the effect of Paxlovid treatment within 5 days of COVID-19 index on the risk of severe COVID-19 outcomes within 28 days of COVID-19 index. |
| Setting | 5 | Describe the setting, locations, and relevant dates, including periods of recruitment, exposure, follow-up, and data collection | Methods – Eligibility Criteria, Paragraph 1 | We defined our study period as April 1, 2022 to August 28, 2023, with an index cutoff date of July 31, 2023. |
| Participants | 6 | (*a*) *Cohort study*—Give the eligibility criteria, and the sources and methods of selection of participants. Describe methods of follow-up  *Case-control study*—Give the eligibility criteria, and the sources and methods of case ascertainment and control selection. Give the rationale for the choice of cases and controls  *Cross-sectional study*—Give the eligibility criteria, and the sources and methods of selection of participants | Methods – Eligibility Criteria, Paragraph 1 | To meet the eligibility criteria for the study as per the target trial protocol, we specified the following inclusion criteria: 1) having a documented COVID-19 index date within the study period...  We also specified three exclusion criteria: 1) patients who were hospitalized on or before the COVID-19 index date or date of treatment with Paxlovid…  We followed patients for a 28-day period following their COVID-19 index date. |
|  |  | (*b*) *Cohort study*—For matched studies, give matching criteria and number of exposed and unexposed  *Case-control study*—For matched studies, give matching criteria and the number of controls per case |  |  |
| Variables | 7 | Clearly define all outcomes, exposures, predictors, potential confounders, and effect modifiers. Give diagnostic criteria, if applicable | Methods – Treatment and Outcome, Paragraph 1 | Eligible patients were categorized by their treatment exposure, defined as receiving a Paxlovid prescription within a 5 day grace period of their COVID-19 index date, with controls defined as patients not prescribed Paxlovid in that period.  Our primary outcome was hospitalization at any point during the 28-day follow-up period. We also assessed death and a composite outcome of death or hospitalization as additional outcomes. |
| Data sources/ measurement | 8* | For each variable of interest, give sources of data and details of methods of assessment (measurement). Describe comparability of assessment methods if there is more than one group | Methods – Cloning, Censoring, and Weighting, Paragraph 5 | IPCW covariates included sex, age (binned), race and ethnicity, prior history of individual comorbid conditions captured in the Charlson Comorbidity index, value of the composite Charlson Comorbidity Index (CCI; binned),… |
| Bias | 9 | Describe any efforts to address potential sources of bias | Methods – Cloning, Censoring, and Weighting, Paragraph 2 | Assigning each patient to both treatment strategies that are compatible with their observed data at *t*_0_ eliminates immortal time bias, because the period between *t*_0_ and *t*_A_ counts as both treated and untreated…  Although the clone-censor-weight technique removes immortal time bias, it also introduces selection bias through informative artificial censoring. To adjust for this selection bias, we estimated inverse probability of censoring weights (IPCW). |
| Study size | 10 | Explain how the study size was arrived at | Results, Figure 1, Patient Characteristics, Paragraph 1 | Figure 1  In the study cohort (within our defined study period), a total of 703,647 patients had a valid COVID-19 index date during the study period, of which 206,393 (20.6%) were treated with Paxlovid, and a total of 13,895 (2.0%) patients across the entire study cohort were hospitalized. After applying the eligibility criteria to the patient population and selecting for high-fidelity Paxlovid prescription sites, a total of 34 of 76 study sites were retained. |

Continued on next page

| Quantitative variables | 11 | Explain how quantitative variables were handled in the analyses. If applicable, describe which groupings were chosen and why | Methods – Cloning, Censoring, and Weighting, Paragraph 5 | IPCW covariates included sex, age (binned), race and ethnicity, prior history of individual comorbid conditions captured in the Charlson Comorbidity index, value of the composite Charlson Comorbidity Index (CCI; binned),… |
| --- | --- | --- | --- | --- |
| Statistical methods | 12 | (*a*) Describe all statistical methods, including those used to control for confounding | Methods – Descriptive Statistics, Paragraph 1; Methods – Cloning, Censoring, and Weighting, Paragraphs 1-5 | First, we applied two-sided Chi-squared tests to examine the distribution of Paxlovid treatment across two covariates…  Next, we used the potential outcomes framework to estimate the effect of Paxlovid treatment on hospitalization. We applied the clone-censor-weight technique… |
|  |  | (*b*) Describe any methods used to examine subgroups and interactions | Methods – Vaccination-adjusted Subanalysis and Stratified Analysis, Paragraphs 1 and 4;  Methods – Age-Stratified Analysis, Paragraph 1 | We conducted a vaccination-subanalysis in a subset of sites with high-quality vaccination data. We used the same method applied to the primary analysis described above with an additional indicator of whether or not the patient was fully vaccinated at least two weeks prior to index included as a covariate…  We also conducted a stratified analysis to examine effect heterogeneity by vaccination status…  We conducted a second stratified analysis to examine effect heterogeneity across three strata of patient age at COVID-19 index: 18-49 years, 50-64 years, and 65+ years… |
|  |  | (*c*) Explain how missing data were addressed | Methods – Cloning, Censoring, and Weighting, Paragraph 5 | CCI was coded as missing when no condition exposures were present in N3C prior to index. CWBI was coded as missing when patient ZIP code was not reported. |
|  |  | (*d*) *Cohort study*—If applicable, explain how loss to follow-up was addressed  *Case-control study*—If applicable, explain how matching of cases and controls was addressed  *Cross-sectional study*—If applicable, describe analytical methods taking account of sampling strategy | Methods – Cloning, Censoring, and Weighting, Paragraph 3 | Next, we applied artificial censoring to ensure that patients and clones follow their assigned treatment strategy after t0. Clones were censored at the time of their true patient counterpart’s tA. Therefore, clones of treated patients were censored from the control arm at the date of their true counterpart’s treatment. Clones of control patients were censored from the treated arm at day 5, when the grace period ended. In addition to this artificial censoring, for the hospitalization outcome, we treated death as a censoring event if it occurred post-treatment assignment, pre-outcome, within the study period. |
|  |  | (*e*) Describe any sensitivity analyses | Methods – Vaccination-adjusted Subanalysis and Stratified Analysis, Paragraphs 1 and 4;  Methods – Age-Stratified Analysis, Paragraph 1 | We conducted a vaccination-subanalysis in a subset of sites with high-quality vaccination data. We used the same method applied to the primary analysis described above with an additional indicator of whether or not the patient was fully vaccinated at least two weeks prior to index included as a covariate…  We also conducted a stratified analysis to examine effect heterogeneity by vaccination status…  We conducted a second stratified analysis to examine effect heterogeneity across three strata of patient age at COVID-19 index: 18-49 years, 50-64 years, and 65+ years… |
| Results | | | | |
| Participants | 13* | (a) Report numbers of individuals at each stage of study—eg numbers potentially eligible, examined for eligibility, confirmed eligible, included in the study, completing follow-up, and analysed | Results – Figure 1 | Study Cohort and Flow of Emulated Trial |
|  |  | (b) Give reasons for non-participation at each stage |  |  |
|  |  | (c) Consider use of a flow diagram |  |  |
| Descriptive data | 14* | (a) Give characteristics of study participants (eg demographic, clinical, social) and information on exposures and potential confounders | Results – Table 2 | Baseline Population Characteristics |
|  |  | (b) Indicate number of participants with missing data for each variable of interest |  |  |
|  |  | (c) *Cohort study*—Summarise follow-up time (eg, average and total amount) |  |  |
| Outcome data | 15* | *Cohort study*—Report numbers of outcome events or summary measures over time | Results – Table 2 | Baseline Population Characteristics |
|  |  | *Case-control study—*Report numbers in each exposure category, or summary measures of exposure |  |  |
|  |  | *Cross-sectional study—*Report numbers of outcome events or summary measures |  |  |
| Main results | 16 | (*a*) Give unadjusted estimates and, if applicable, confounder-adjusted estimates and their precision (eg, 95% confidence interval). Make clear which confounders were adjusted for and why they were included | Results- Table 3 | Estimated Cumulative Incidence, Relative Risks, and Absolute Risk associated with treatment with Paxlovid across all analyses |
|  |  | (*b*) Report category boundaries when continuous variables were categorized | Results- Table 2 | Baseline Population Characteristics |
|  |  | (*c*) If relevant, consider translating estimates of relative risk into absolute risk for a meaningful time period | Results- Table 3 | Estimated Cumulative Incidence, Relative Risks, and Absolute Risk associated with treatment with Paxlovid across all analyses |

Continued on next page

| Other analyses | 17 | Report other analyses done—eg analyses of subgroups and interactions, and sensitivity analyses | Results – Effect of Paxlovid by COVID-19 Vaccination Status, Paragraph 1;  Effect of Paxlovid by Age;  Table 3 | | For our subgroup analysis estimating the effect of Paxlovid on hospitalization, we examined both vaccination status and age groups. The vaccination …  In our age-stratified analysis, we observed varying effects of Paxlovid across different age groups. Notably, while Paxlovid…  Table 3: Estimated Cumulative Incidence, Relative Risks, and Absolute Risk associated with treatment with Paxlovid across all analyses |
| --- | --- | --- | --- | --- | --- |
| Discussion | | | | | |
| Key results | 18 | Summarise key results with reference to study objectives | | Discussion – Paragraph 1,  Paragraph 3,  Paragraph 4,  Paragraph 5 | In this target trial emulation using the N3C database, Paxlovid treatment within 5 days of a COVID-19 diagnosis or positive SARS-CoV-2 test reduced the risk of hospitalization by 39%, death by 61%, and hospitalization or death by 40%.  In a subcohort with reliable vaccination data, we found that Paxlovid treatment reduced the absolute risk of hospitalization by the same amount (0.7 percentage points), regardless of vaccination status.  In an age-stratified analysis, we found a larger absolute risk reduction for patients aged 65+ (1.3 percentage points) compared to patients ages 18-49 and 50-64 (0.7 percentage points for both groups).  We also found large differences in Paxlovid treatment rates by race, ethnicity, and CWBI. |
| Limitations | 19 | Discuss limitations of the study, taking into account sources of potential bias or imprecision. Discuss both direction and magnitude of any potential bias | | Discussion, Paragraph 7 | This study also has several limitations. First… |
| Interpretation | 20 | Give a cautious overall interpretation of results considering objectives, limitations, multiplicity of analyses, results from similar studies, and other relevant evidence | | Discussion, Paragraph 8 | We found that Paxlovid treatment within 5 days of a COVID-19 diagnosis or positive test reduced the risk of hospitalization by 39%. Broadly speaking, our findings are consistent with the evidence base: Paxlovid is effective at preventing severe COVID-19 outcomes, but it is less effective in real-world settings than in early clinical trials… |
| Generalisability | 21 | Discuss the generalisability (external validity) of the study results | | Discussion, Paragraph 7 | Fifth, this study’s eligibility criteria include indication for on-label Paxlovid treatment (i.e., at risk for developing severe COVID-19 due to the presence of one or more risk factors). Therefore, results can only be generalized to a high-risk population. |
| Other information | |  | | | |
| Funding | 22 | Give the source of funding and the role of the funders for the present study and, if applicable, for the original study on which the present article is based | | Acknowledgements, Paragraph 2 (N3C Attribution) | N3C Attribution – The analyses described in this publication were conducted with data or… |

*Give information separately for cases and controls in case-control studies and, if applicable, for exposed and unexposed groups in cohort and cross-sectional studies.

**Note:** An Explanation and Elaboration article discusses each checklist item and gives methodological background and published examples of transparent reporting. The STROBE checklist is best used in conjunction with this article (freely available on the Web sites of PLoS Medicine at http://www.plosmedicine.org/, Annals of Internal Medicine at http://www.annals.org/, and Epidemiology at http://www.epidem.com/). Information on the STROBE Initiative is available at www.strobe-statement.org.
