# Supplementary material for: Effect of nirmatrelvir/ritonavir (Paxlovid) on hospitalization among adults with COVID-19: An electronic health record-based target trial emulation from N3C
Source: PLoS Med. 2025 Jan 17;22(1):e1004493. doi: 10.1371/journal.pmed.1004493 (PMC11790232; doi:10.1371/journal.pmed.1004493)
Supplement: S1 Fig — (DOCX) [file pmed.1004493.s002.docx]

# **S1 Fig**

Proportion of individuals in base population stratified by Race and Ethnicity, and ZIP code-level Community Wellbeing Index (CWBI)

**
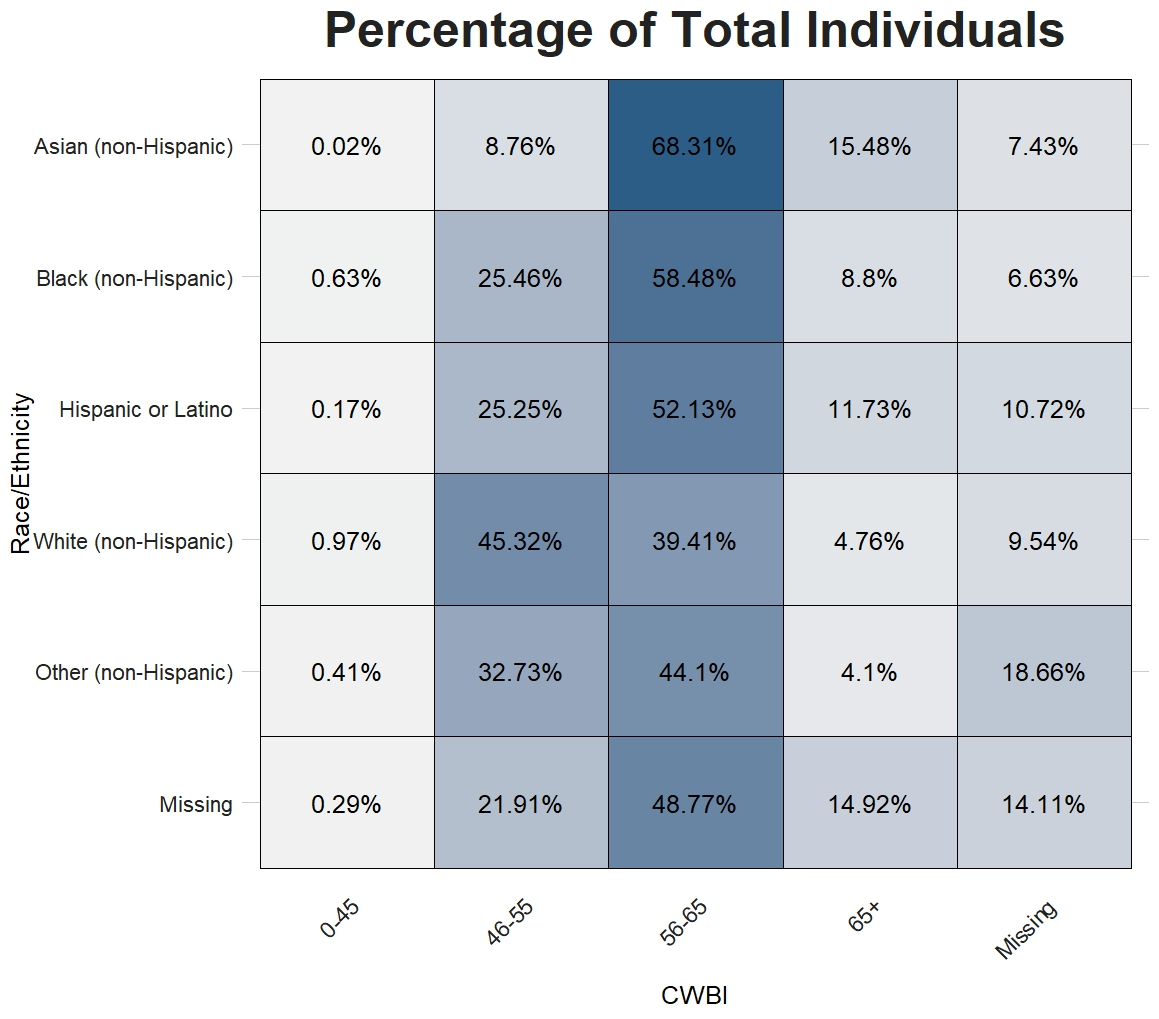
**
